# [Retracted] Potentiation of the antitumor activity of adriamycin against osteosarcoma by cannabinoid WIN‑55,212‑2

**DOI:** 10.3892/ol.2023.14183

**Published:** 2023-12-11

**Authors:** Feng Niu, Song Zhao, Chang-Yan Xu, Hui Sha, Gui-Bin Bi, Lin Chen, Long Ye, Ping Gong, Tian-Hong Nie

Oncol Lett 10: 2415–2421, 2015; DOI: 10.3892/ol.2015.3525

Following the publication of this paper, it was drawn to the Editor's attention by a concerned reader that the scratch-wound assay data shown in Fig. 2 on p. 2418 in this paper contained a number of anomalous features that could not readily be explained by chance alone, or through the coincidental placement of similar-looking data. Consequently, the Editor of *Oncology Letters* has determined that this paper should be retracted from the Journal. The authors were asked for an explanation to account for these concerns, but the Editorial Office did not receive a reply. The Editor apologizes to the readership for any inconvenience caused.

